# Population Genetic Analysis of *Phytophthora colocasiae* from Taro in Japan Using SSR Markers

**DOI:** 10.3390/jof9040391

**Published:** 2023-03-23

**Authors:** Jing Zhang, Ayaka Hieno, Kayoko Otsubo, Wenzhuo Feng, Koji Kageyama

**Affiliations:** 1 Key Laboratory of Agricultural Microbiology, College of Agriculture, Guizhou University, Guiyang 550025, China; 2 River Basin Research Center, Gifu University, 1-1 Yanagido, Gifu 501–1193, Japan

**Keywords:** *Phytophthora colocasiae*, taro leaf blight, genetic diversity, population structure, mating type, polyploidy, SSR

## Abstract

*Phytophthora colocasiae* is an important pathogen that causes great economic losses in taro production in tropical and subtropical regions, especially in Japan. Understanding the genetic variations in *P. colocasiae* populations and their transmission patterns in Japan is essential for effective disease control. Here, the genetic diversity of 358 *P. colocasiae* isolates, including 348 from Japan, 7 from China, and 3 from Indonesia, was assessed using 11 simple sequence repeat (SSR) primer pairs with high polymorphism. The phylogenetic tree of the SSR locus showed that the isolates from Japan could be divided into 14 groups, with group A being the dominant group. Among foreign isolates, only six from mainland China were similar to those from Japan and clustered in groups B and E. Analysis of molecular variance (AMOVA), principal components analysis (PCA), and cluster analysis (K = 3) results revealed a moderate level of genetic diversity, mainly within individuals. Populations showed high heterozygosity, a lack of regional differentiation, and frequent gene flow. Analysis of mating types and ploidy levels revealed that A2 and self-fertile (SF) A2 types and tetraploids were dominant across populations. Explanations and hypotheses for the results can provide more effective strategies for disease management of taro leaf blight.

## 1. Introduction

*Phytophthora colocasiae* Raciborski is a major oomycete pathogen that threatens the sustainable production of taro (*Colocasia esculenta* (L.) Schott). Since its discovery in Java in 1900, this pathogen has spread rapidly throughout tropical and subtropical regions, including southeast Asia, many Pacific islands, and portions of Africa and Oceania [1,2]. The pathogen causes taro leaf blight and infects even taro corms, which poses a severe risk for taro storage and seeding. Taro leaf blight often occurs during hot and humid seasons, causing extensive leaf rot and even plant dieback [3]. In Japan, there have been severe outbreaks of the disease since 2014. The epidemic then quickly spread to the prefectures of Kagoshima, Miyazaki, and Ehime [4]. Recently, the disease has extended to Chiba, Saitama, Fukui, and Gifu and is still spreading. To control this disease and develop appropriate and effective management strategies, an understanding of the geographic relevance and infection routes of the pathogen is urgently needed.

*Phytophthora colocasiae* produces asexual structures, such as zoospores, which can swim and spread in water and infect other host plants [5]. Additionally, sexual oospores are crucial to the life cycle of such pathogens, although most *Phytophthora* species are thought to be heterothallic [6]. Furthermore, another mating type of *Phytophthora* has been increasingly detected, which can reportedly self-fertilize to produce oospores [7,8]. *P. colocasiae* has also been confirmed in our previous studies, and results suggest that five mating types are plainly identified in Japan: heterothallic A1 and A2, as well as self-fertile (SF) A1, A2, and A1/A2 [9]. Such reproductive modes and reproductive structures allow the pathogen to show a high degree of variability and a strong ability to spread. Therefore, the study of its genetic diversity in time and space can further our understanding of intra-species differences, explore its evolutionary history, and lay a solid theoretical foundation for the analysis of its transmission route.

Toxicity identification is a traditional method used to study the genetic diversity of pathogen populations [10], and the analysis of the frequency of pathogenic toxicity genes is important for understanding population structure. Researchers usually judge the toxicity characteristics of pathogens according to the pathogenicity changes in host varieties containing different genes, and then identify their physiological race. However, the traditional method still has some serious shortcomings, as it is easily affected by environmental conditions and host identification, among other factors [11]. Furthermore, the toxicity variation of the pathogen is identified based only on the toxic phenotype, while its genotype remains unknown. Further research on population genetic structure and the rapid development of molecular biology technology has allowed the development of molecular marker techniques based on DNA polymorphisms, providing a very important means for revealing the genetic relationship between populations or individuals in the study of genetic diversity. Molecular markers are extensively distributed in the genome of organisms and are abundant, easy to obtain, convenient to use, and inexpensive. These techniques, such as simple sequence repeats (SSR), have been extensively employed in population genetics investigations of plant pathogenic fungi [12,13,14,15,16,17].

SSR markers (also known as microsatellites) are highly mutable and repetitive sequences, commonly used as genetic markers [18]. Microsatellites have the advantages of multiple alleles, co-dominance, high polymorphism, and only small amounts of DNA [19]. Compared with amplified fragment length polymorphism (AFLP) and random amplified polymorphic DNA (RAPD) markers, SSR markers have higher polymorphic information content and richer genetic diversity [20,21]. SSR markers are frequently employed in conservation genetics to support fundamental genetic research and plant breeding activities [22,23,24]. Indeed, in recent years, SSR markers have been developed for many crops [25,26] and plant pathogens [27]. However, the use of SSR markers in *P. colocasiae*, particularly in Japan, has not been reported. Therefore, this molecular marker technique was used as the main source of genotyping data for this study.

Specifically, the purposes of this study included (i) developing trustworthy SSR markers for *P. colocasiae* population genetic analysis, (ii) determining the predominant genotype of *P. colocasiae*, (iii) calculating the genetic diversity within *P. colocasiae* populations, and (iv) comprehending the modes of *P. colocasiae* transmission throughout Japan.

## 2. Materials and Methods

### 2.1. Isolates and DNA Extraction

A total of 358 isolates of *P. colocasiae* were obtained from Japan, China, and Indonesia (Table S1). Each isolate was collected from diseased taro plants, soil, and river water. A total of 347 isolates from Japan were obtained from the main taro-growing areas of Japan between 2014 and 2020, including Kagoshima, Miyazaki, Ehime, Chiba, Saitama, Fukui, and Gifu Prefectures [9]. The other 11 isolates, one from Okinawa and 10 from other countries, were obtained from the collection of the River Basin Research Centre, Gifu University, Japan. Each isolate was recovered using selective cornmeal agar supplemented with nystatin, ampicillin, rifampicin, and miconazole (NARM) [28] and confirmed by morphological characteristics. The isolates were kept on corn meal agar (CMA) at 20 °C in the dark. PrepMan Ultra Reagent (Applied Biosystems, Foster City, CA, USA) was used to extract genomic DNA from mycelia, as described by Baten et al. [29].

### 2.2. Mating-Type Determination

The mating types of most isolates were identified in our previous study [9], and the results are shown in Table S1. The remaining strains were tested as previously described [9].

### 2.3. SSR Markers Development and PCR Reactions

The entire genome sequence of *P. colocasiae* was checked for SSR motifs using the Tandem Repeat Finder [30]. SSR markers were selected based on repeats, and primers were designed manually in conserved regions. The best primers were selected after their specificity was tested by Primer BLAST, and the absence of dimer potential was confirmed with NetPrimer (http://www.premierbiosoft.com/NetPrimer/AnalyzePrimer.jsp; (accessed on 4 September 2018)). All 35 primer pairs were then tested using a variety of isolates from various geographic locations to choose the primers that consistently amplified the repeat patterns, had the predicted size after electrophoresis, and revealed numerous repetitions by sequencing.

The PCR reaction mixture had a total volume of 25 μL containing 2.5 µL of 10 × PCR Buffer (100 mM, pH 8.3 Tris-HCl, 15 mM MgCl_2_ and 500 mM KCl), 2.0 µL of 2.5 mM dNTPs, 0.2 µL of 25 µM of each forward and reverse primer, 0.01 mg bovine serum albumin (Sigma-Aldrich, Tokyo, Japan), 0.125 µL rTaq DNA polymerase (Takara Bio, Shiga, Japan), 1 µL of template DNA (1 ng/µL), and 15.875 µL ddH_2_O. The amplification was carried out using a Gene Amp PCR system 2700 thermal cycler (ABI, Tokyo, Japan) under the following conditions: pre-denaturation at 94 °C for 2 min; and then 35 cycles of denaturation at 94 °C for 30 s, annealing at optimum temperature (see Table 1) for 30 s, and extension at 72 °C for 30 s; and a final extension at 72 °C for 10 min. The amplification products were separated on 2% agarose gel, stained with GelRed, and photographed under UV light.

The remaining PCR products were recovered and used for sequence analysis according to a process outlined by Masanto et al. [31]. The TOPO TA cloning kit was used to clone the amplified fragments (Invitrogen, Carlsbad, CA, USA). More than 18 *E. coli* recombinants were chosen for colony PCR using the primer pair, M13M4 (GTTTTCCCAGTCACGAC) and M13Rv (CAGGAAACAGCTATGAC). Approximately 12 effective PCR products were purified utilizing the ExoSAP-IT kit as directed by the manufacturer’s instructions (Affymetrix, Santa Clara, CA, USA). The purified solutions were cycled once again using a Big Dye Terminator ver 3.1 Cycle Sequencing Kit (Applied Biosystems, Foster City, CA, USA) under the single primer M13M4, and then ethanol-precipitated in the dark to make the final sequence using an ABI 3100 or ABI 3130 Genetic Analyzer (Applied Biosystems). The frequency and diversity of the repetitive sequences were analyzed to select feasible primers for subsequent genotyping. The process is shown in Figure S1.

### 2.4. SSR Genotyping

In all, 358 isolates of *P. colocasiae*, listed in Table S1, were used to test each primer set. The developed primer sets were paired based on their amplified fragments that differed significantly and were separately labeled at the 5′ end of the primer with the fluorescent dye FAM or HEX. The total genomic DNA of the isolates was amplified using each primer set under the conditions described above. To analyze each PCR fragment, a 10-µL reaction mixture was prepared and measured using an ABI 3100 or ABI 3130 Genetic Analyzer. The mixture consisted of 1 µL of Fam-labeled PCR product, 1 µL of another HEX-labelled PCR product, 1 µL of 1:4 diluted 500LIZ specification standard, and 10 µL of Hi-Di formamide. In some special cases, such as fragments of similar size, FAM- or HEX-labeled PCR products are best analyzed separately to avoid interference. Finally, the band peaks and sizes of all SSR fragments were recorded using GeneMapper Software version 4.1.

### 2.5. Data and Population Structure Analysis

The effective number of alleles, observed heterozygosity (Ho), expected heterozygosity (He), polymorphism information content (PIC), and the analysis of molecular variance (AMOVA) were calculated using PolyGene V1.4 software [32,33]. The estimation of ploidy level was conducted by the R package “polysat” version 1.7–5 with R Version 3.6.2 [34,35].

In the phylogenetic analysis, a neighbor-joining tree with bootstrap values calculated from 1000 replicates was constructed using PAUP* version 4.0a (build 169) previously described by Hattori et al. [36]. The generated trees were visualized using FigTree, version 1.4.4. Principal components analysis (PCA) plots with Nei’s standard genetic distance were generated by PolyGene, while PCA plots of Bruvo distance and Lynch distance were generated by R “polysat”. A PCA plot for the estimated tetraploid isolates was created using the Bruvo distance calculated considering ploidy (*n* = 4) and allele frequency. Population structure analysis was performed using the Bayesian clustering method in PolyGene. Each population was divided into subpopulations according to the estimated ploidy level, and K = 1–5 was calculated independently. Then, one of the results for K = 3 was selected manually. Hierarchical clustering of the population of the estimated tetraploid isolates was performed using PolyGene. Finally, a dendrogram was generated using the UPGMA clustering method based on Nei’s standard genetic distances.

## 3. Results

### 3.1. SSR Markers Development and Polymorphism

The whole genome sequence of *P. colocasiae* was analyzed using Tandem Repeat Finder, and 35 primers were selected to amplify the microsatellite loci. Three isolates from Japan and India with different mating types (EPC201522, EPC2017Ko1, and 56TAROKDTL) were tested, and 11 loci with multiple alleles were selected for subsequent SSR marker studies (TAT_66, CCT_4368, CTT_270, CTT_1936, GCT_5986, TCC_502, CTA_421, TAG_1296, GA_106, TCC_1066, and AGAC_2040). A 7 bp tail (GTGTCTT) was added to the reverse primer of the 11 SSR primers to promote 3′ non-templated nucleotide addition, and 358 *P. colocasiae* isolates (Table S1) were amplified by PCR. Electrophoresis results showed 11 pairs of SSR primers that specifically amplified 11 microsatellite loci.

The amplified bands ranged from 78–227 bp in size (Table 1). A total of 39 alleles were detected in 385 *P. colocasiae* isolates, and the number of alleles ranged from 2–5, with an average of 3.55 per locus. The effective number of alleles ranged from 1.01–4.00 (average 2.13). The mean Ho was 0.52, whereas the maximum (1.00) and the minimum (0.004) were found at the GCT_5986 and AGAC_2040 loci, respectively. The GCT_5986 locus had the highest He detection rate (0.75), whereas the AGAC_2040 and GA_106 loci had the lowest (0.01, respectively). Several loci were polymorphic, with a mean PIC of 0.37, ranging from 0.01 (AGAC_2040 and GA_106) to 0.70 (GCT_5986). The PIC value indicated that the locus was reasonably informative (0.5 < PIC > 0.25) and could provide reliable genetic information for this study (Table 1).

### 3.2. Mating-Type Diversity

All 358 isolates, including those determined in a previous study, were counted as mating types [9]. Four mating types, self-fertile (SF) A2 and A1/A2 types, and heterothallic A1 and A2 types, were identified by observing the sexual structures (oogonia, antheridia, and oospores) in both the single cultures and the reactionary zones between paired colonies. Of the 358 isolates, there were 13 heterothallic A1 and 224 heterothallic A2 types. The remaining 121 isolates were classified as SF due to the production of sexual structures in single cultures but were further divided into 115 SF A2 and 6 A1/A2 isolates that also produced abundant sexual structures in the reaction zones in cultures paired with the A1 type strain and both strains of the A1 and A2 types (Table S1).

### 3.3. Population Genetic Differentiation

To evaluate the differences and contributions of genetic diversity between or among populations, some statistical methods were fully utilized to calculate AMOVA, such as homoploidy, anisoploidy, the maximum likelihood method, and weighted genotypes [37]. Here, we used the weighted genotype method to perform the AMOVA in the POLYGENE program because it represents the least deviated genetic distance and is considered suitable for polyploid phenotypic datasets [33]. Moreover, the sum of squares (SS) value to measure variation was calculated based on the *infinity allele model* (IAM) [37] or the *stepwise mutation model* (SMM) genetic distance [38].

Our AMOVA results (Table 2) showed that individuals shared 116.19% (IAM) or 117.42% (SMM) of the total genetic variation; no fraction was partitioned among individuals within populations, while a negative component of −16.19% (IAM) and −17.42% (SMM) of the total genetic variation occurred among populations. In addition, for both IAM and SMM, there was a negative level of inbreeding with the total population (*F_IT_* = −0.162 and −0.174), a negative level of inbreeding between individuals within populations (*F_IS_* = −0.19 and −0.196), and low differentiation among populations (*F_ST_* = 0.024 and 0.018). The value (0.05 < *F_ST_* > 0) indicated that there was essentially no genetic variation among populations because as *F_ST_* values decline, genetic variation between populations becomes less significant [39], while most genetic variation comes from within populations. The observed heterozygosity was higher than expected, yielding negative values for *F_IT_* and *F_IS_*, and indicated heterozygosity within the population.

**Figure 1:**
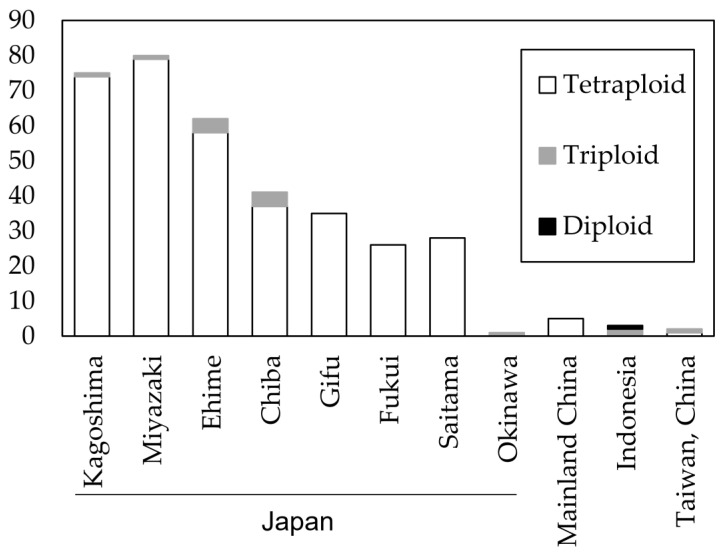
Number of isolates with their ploidy level in each of the 11 populations estimated by the R package “polysat”.

The ploidy levels of the individuals were determined based on the maximum and mean number of alleles per locus for all samples and across all loci using the R package “polysat” [34,40]. An individual was tetraploid when at least one of the 11 sites had 4 alleles, triploid when the maximum number of alleles was 3, and diploid when there were only 2 alleles. The levels of ploidy in different populations were counted, and the results showed that most isolates from Japan and mainland China were tetraploid, while a few triploid isolates existed in populations of Japan, Indonesia, and Taiwan, China, and diploidy was only found in the P6317 isolate from Indonesia (Figure 1).

### 3.4. Clustering and Population Genetic Structure

**Figure 2:**
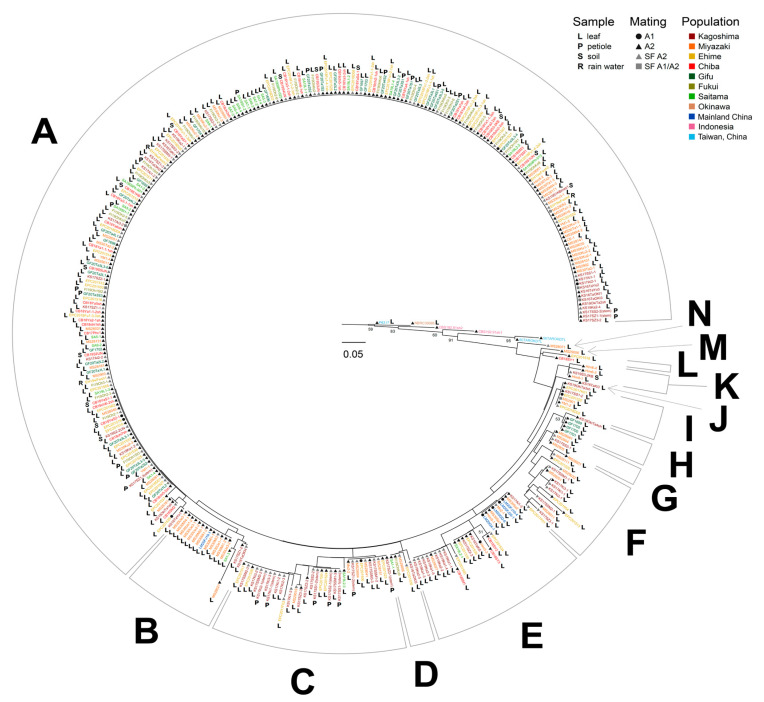
Multilocus microsatellite phylogenetic tree of 358 isolates of *Phytophthora colocasiae*. Numbers above branches are bootstrap values (>50). Genotype groups are labeled with A to N. SF: self-fertilization.

The neighbor-joining dendrogram was used to analyze all 358 isolates and grouped the isolates from 7 populations in Japan (except for the isolate from Okinawa) into 14 groups from A–N (Figure 2). Three isolates, KS16Taki3, MS19056, and MS28071, were divided into groups J, M, and N, respectively. The other groups contained isolates collected from two or more populations. Group A was the largest cluster, containing 231 isolates from all populations. Groups B, F, and I contained 15, 15, and 7 isolates from the 3 populations, respectively. Group C included 33 isolates from Kagoshima, Miyazaki, Ehime, and Saitama. Groups D and E contained 5 and 23 isolates from 5 populations, respectively. Of the remaining 15 isolates, 4, 5, 4 and 2 isolates from the 2 types of populations were divided into groups G, H, K, and L, respectively. Such complex clusters were discovered in the populations, demonstrating the genetic variability within the individuals in each population.

**Figure 3:**
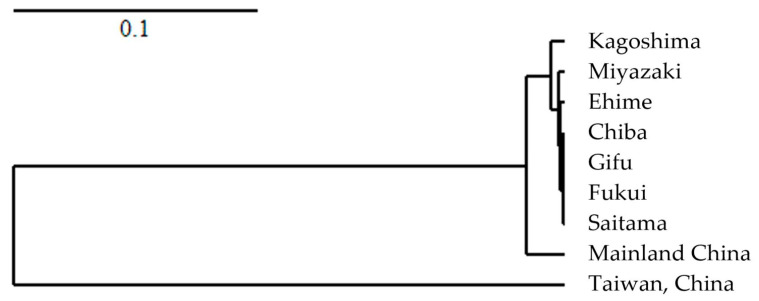
Hierarchical clustering for tetraploid isolates in each population calculated using PolyGene software. Dendrogram generated with the UPGMA clustering method based on Nei’s standard genetic distances.

In addition, nine populations containing tetraploid strains were analyzed using the UPGMA clustering method. The results showed that populations from central and northern Japan were consistent, and their genetic relationships were similar to those from southern Japan. However, the populations from Japan are quite different from those from China, especially the population from Taiwan, probably due to the small number and long history of population surveys in Taiwan (Figure 3).

**Figure 4:**
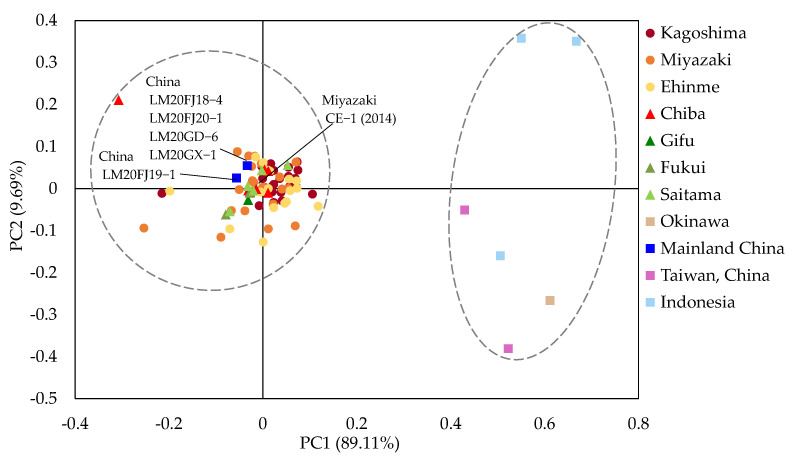
PCA plot of Nei’s standard genetic distance calculated by PolyGene software. Circles show the grouping of the isolates.

Multivariate analysis using PCA is frequently used to identify genetic variation patterns among populations. As Figure 4 shows, based on Nei’s standard genetic distance, the *P. colocasiae* isolates were divided into two major groups. The first and second axis explained 89.11% and 9.69% of the overall variation, respectively, for a total explanation of 98.80%. One group encompassed all isolates from Japan, except the one from Okinawa and the isolates from mainland China. Meanwhile, the other group contained isolates from Indonesia, Okinawa, and Taiwan. The PCA outputs were consistent with those obtained from the phylogenetic tree. Moreover, the other two genetic distances of Bruvo and Lynch were also used for PCA analysis for all isolates, and only the Bruvo distance was calculated to consider the ploidy and allele frequency for tetraploid isolates (Figure S2).

**Figure 5:**
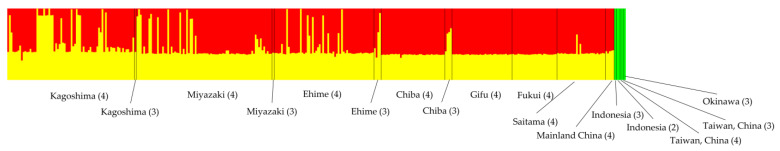
Bayesian clustering in each of the 11 populations calculated by PolyGene software, K = 3 (red, yellow, and green). The number in () after the population is the ploidy estimated by the R package “polysat”.

Cluster analysis showed that the optimal number of genotypic clusters represented within the data was K = 3, which was applied to all 11 geographic populations (Figure 5). All populations had three genetic sources. The isolates from Okinawa, Indonesia, and Taiwan, which only had green bars, were considered as one cluster and differed from the others. In addition, the isolates from Japan and mainland China had both red and yellow bars, indicating that the isolates shared a common genetic basis and were assigned to the two clusters. One population had a slightly higher proportion of red bars, including all populations. Another cluster was identified and contained isolates from Kagoshima, Miyazaki, Ehime, Chiba, and Saitama due to the high percentage of yellow bars. Finally, we distinguished the triploid and tetraploid strains, and the results revealed no clear correlation between ploidy level and gene source.

## 4. Discussion

With the increasing transmission capacity of *P. colocasiae* and its resistance becoming increasingly prominent in planting ecology, taro leaf blight has become more common and difficult to control. Further, pathogens with a mixed reproductive system, large effective population sizes, and high potential for genotype flow are thought to cause serious damage to host resistance genes [41]. Therefore, large-scale evaluation of the genetic diversity of the pathogen is vital for precautionary strategy design. Currently, research on the genetic diversity of *P. colocasiae* is limited. SSR markers with the advantages of co-dominance, high polymorphism, reliability, and repeatability can reveal polymorphisms among closely related isolates and have been widely used to characterize the population structure of *Phytophthora* [16,42]. In this study, the genetic diversity and population structure of 358 isolates, including 348 isolates from 8 regions in Japan, 7 isolates from China, and 3 isolates from Indonesia, were analyzed using 11 pairs of SSR primers. Here, 2–5 alleles were present at each locus, which was lower than reported in previous studies of *P. nicotianae* with 4–11 alleles and *Phytopythium helicoides* with 10–21 alleles at each locus [43,44]. We hypothesized that this is because the host range of this pathogen (only host of taro) is narrower than those of the other 2 pathogens (*P. nicotianae* with more than 250 hosts [45] and *P. helicoides* with more than 27 hosts (National Fungus Collections Fungus-Host Distribution Database)). Nevertheless, PIC and gene diversity analyses showed that the *P. colocasiae* isolates had abundant alleles and gene diversity.

All isolates were analyzed using the topology of a neighbor-joining tree. The results showed that the isolates from Japan differed from those from Indonesia and Taiwan. It is possible that the isolates obtained from Indonesia and Taiwan were quite old; nevertheless, the isolates collected from Indonesia in 2017 were quite different. These results indicate that gene exchange between Indonesia and Japan has seemingly been limited. In addition, the genotypes of the pathogen changed greatly with time when comparing the isolates from the last and this century. Isolates from Japan were subdivided into 14 groups. Group A (65%) was the dominant genotype and was distributed across all populations under study, showing no sign of decrease from 2014 to 2020 and remaining the predominant group (Table S2). The genotypes from southern Japan were much more complex than those from central and northern Japan; therefore, it was concluded that the pathogen was likely to flow and spread from south to north, which was also consistent with the time sequence of disease occurrence. In addition, the isolates from mainland China were divided into E and B genotypes and were found to belong to the same subclade as isolates from nearby Kagoshima and Miyazaki (Figure 2), suggesting that the pathogen has experienced a certain extent of gene exchange between the two regions. However, these two genotypes do not appear elsewhere in Japan. Therefore, we concluded that the genotypes may have drifted from China to Kagoshima and Miyazaki, but their spread was limited owing to the influence of the major A genotype.

Our results indicate that all populations in Japan are genetically similar among them. First, the results of SSR genotyping showed that group A constituted the majority in each population. Second, AMOVA results indicated that the variance within populations was more than 100% and that among populations was negative, and as the *F_ST_* value was lower than 0.05, the largest variation was within populations. Third, genetic distance analysis of all tetraploid strains showed that populations in northern and central Japan were slightly, but not significantly, different from those in southern Japan. This is consistent with the SSR genotyping results. The former genotype was relatively simple, whereas the latter was relatively mixed. Fourth, PCA results revealed that no clusters were developed in Japan, indicating no correlation between genetic characteristics and geographical location. Finally, this conclusion was supported by cluster analysis of all populations with K = 3.

Oospores can reportedly contribute to the genetic diversity of *Phytophthora* pathogens [46]. Consistent with previous reports [9], here, we found that A2 and SF A2 mating types were better adapted and dominant (more than 94%) in Japan. Having complex mating types of A1, A2, SF A2, and SF, the A1/A2 ratio calculated suggests that sexual reproduction via producing oospores is possible. In addition, we found that mating types were much more complex in southern Japan than in central or northern Japan (Table S3), supporting the previous hypothesis that the pathogen has spread from south to north. Further, for all populations analyzed in this study, the observed heterozygosity was higher than expected, which yielded negative values for *F_IT_* and *F_IS_*, indicating an obvious heterozygote surplus. Thus, although sexual reproduction is possible in Japan, genetic variation within populations is mainly caused by random mutations in asexual reproduction rather than by sexual recombination.

In addition, our analysis of chromosome ploidy level showed that most of the isolates were tetraploid and only a few were triploid, although *Phytophthora* species have been traditionally considered diploid. To date, the cause of polyploidy has not been clearly explained. Polyploid production is considered long-established rather than random and is thought to be related to environmental conditions, as harsh or disturbed environments are more conducive to adaptation [47]. In this investigation, we also found that tetraploids were prevalent, and the triploid strain was only found in the eastern coastal regions of Japan and Indonesia (Table S4). Nevertheless, the relationship between polyploidy generation and gene or environmental effects warrants further study.

## 5. Conclusions

Eleven SSR loci were analyzed in *P. colocasiae* isolates. Population genetic information showed that the isolates from Japan could be divided into 14 groups, with group A being dominant. A relatively high level of genetic diversity was revealed, mainly within individuals. In addition, there is a lack of regional differentiation in Japanese populations, with frequent gene flow, most likely from the southern to the central and northern regions of the country. The A2 and SF A2 mating types and tetraploids were common in all populations studied. Sexual reproduction and its polyploid characteristics make *P. colocasiae* more likely to survive in the environment and promote the development of genetic diversity. While the original source of *P. colocasiae* in Japan was not identified in this study, this article improves the understanding of *P. colocasiae* gene flow, and provides valuable information for better control of taro leaf blight in Japan.

## Figures and Tables

**Table 1:** Sequences of SSR Primers and Characteristics of Polymorphisms.

No.	Markers	Size Range (bp)	Number of Alleles	Effective Number of Alleles	Ho	He	PIC	Sequence (5′-3′)		Dye	Annealing Temperature (°C)
								Forward Primer	Reverse Primer		
1	TAT_66	160–172	4	2.04	0.57	0.51	0.39	TTGCTAAAGCGCAGATTACGC	GTGTCTTACAGTGCTGCCATCCTACTC	HEX	60
2	CCT_4368	198–222	4	3.00	0.81	0.67	0.59	TCAGCGTGGGTATGTAGTCC	GTGTCTTATGATGGTGACGCAGAGGAA	HEX	63
3	CTT_270	129–153	3	2.08	0.60	0.52	0.40	GCCACGAATAGACGACAGTC	GTGTCTTGCAACTTTACCTGGGGTTGC	FAM	63
4	CTT_1936	128–134	2	1.94	0.50	0.49	0.37	TCTACTGTAACGTCCGTCGC	GTGTCTTATCTCCAGTGCCGAAGAGTC	FAM	60
5	GCT_5986	170–182	4	4.00	1.00	0.75	0.70	CGCTTAGACTTGCGACTACG	GTGTCTTTCCAGAAGACGGGAAACGAC	HEX	60
6	TCC_502	162–168	2	2.00	0.56	0.50	0.38	TCAGCGTGGGTATGTAGTCC	GTGTCTTGCGTATTAAAGCGGACAGGG	FAM	63
7	CTA_421	212–227	5	2.12	0.56	0.53	0.42	CGCTTTGTTGAGTTGGACGA	GTGTCTTTCCAATCCGATCACCACCAA	FAM	63
8	TAG_1296	168–180	4	2.16	0.55	0.54	0.44	ACAGCCATCCAACCATGTAA	GTGTCTTACACTCACACCAAAGTAACGC	HEX	63
9	GA_106	90–100	5	1.01	0.012	0.01	0.01	GCTATTGTCTTACACAGACACG	GTGTCTTGAAGCCCATCCACCTAATGG	FAM	58
10	TCC_1066	78–103	4	2.02	0.57	0.51	0.38	GCCACGAATAGACGACAGTC	GTGTCTTGGGAAGCGACATGGAAGAAG	FAM	60
11	AGAC_2040	213–217	2	1.01	0.004	0.01	0.01	GATGGGAGAAAAAGGTGTCG	GTGTCTTGAGATGTGCTCATCCCATTC	HEX	58
Mean			3.55	2.13	0.52	0.46	0.37				

Ho: observed heterozygosity, He: expected heterozygosity, PIC: polymorphism information content. The effective number of alleles, Ho, He, and PIC, were calculated using PolyGene software. Reverse primers included a 7-bp tail (GTGTCTT) to promote 3′ non-templated nucleotide addition.

**Table 2:** Analysis of molecular variance (AMOVA) in *Phytophthora colocasiae* based on 11 SSR loci.

Partitioning	d.f.	SS	MS	Var	% Var	*F-Statistics*
IAM						
Within individuals	1058	3101.19	2.931	2.931	116.191	*F_IT_* = −0.162
Among individuals within populations	341	363.364	1.066	0	0	*F_IS_* = −0.19
Among populations	16	93.471	5.842	−0.408	−16.191	*F_ST_* = 0.024
Total	1415	3558.025	2.515	2.523	100	
SMM						
Within Individuals	1058	348,136.087	329.051	329.1	117.422	*F_IT_* = −0.174
Among individuals within populations	341	39,142.098	114.786	0	0	*F_IS_* = −0.196
Among populations	16	8323.266	520.204	−48.82	−17.422	*F_ST_* = 0.018
Total	1415	395,601.451	279.577	280.2	100	

AMOVA was performed by PolyGene software. d.f.: degrees of freedom, SS: sum of squares, MS: mean squares, Var: variance components, % var: percentage of variance, *F_IT_*: the fixation index with respect to the total population, *F_IS_*: the fixation index of individuals within populations, and *F_ST_*: the proportion of genetic differentiation.

## Data Availability

The study did not report any data.

## Supplementary Materials

The following supporting information can be downloaded at: https://www.mdpi.com/article/10.3390/jof9040391/s1, Figure S1: Schematic representation of SSR marker development; Figure S2: PCA plot generated by the R package “polysat”; Table S1: List of the tested isolates of *Phytophthora colocasiae*; Table S2: Genotypes in each population from 2014 to 2020; Table S3: Mating types of *Phytophthora colocasiae* and their corresponding genotypes in each population; Table S4: Ploidy level of *Phytophthora colocasiae* and their corresponding genotypes in each population.
