# Supplementary material for: Population Genetic Analysis of Phytophthora colocasiae from Taro in Japan Using SSR Markers
Source: J Fungi (Basel). 2023 Mar 23;9(4):391. doi: 10.3390/jof9040391 (PMC10145753; doi:10.3390/jof9040391)
Supplement: Supplementary file 1 [file jof-09-00391-s001.zip › Figures.pdf]

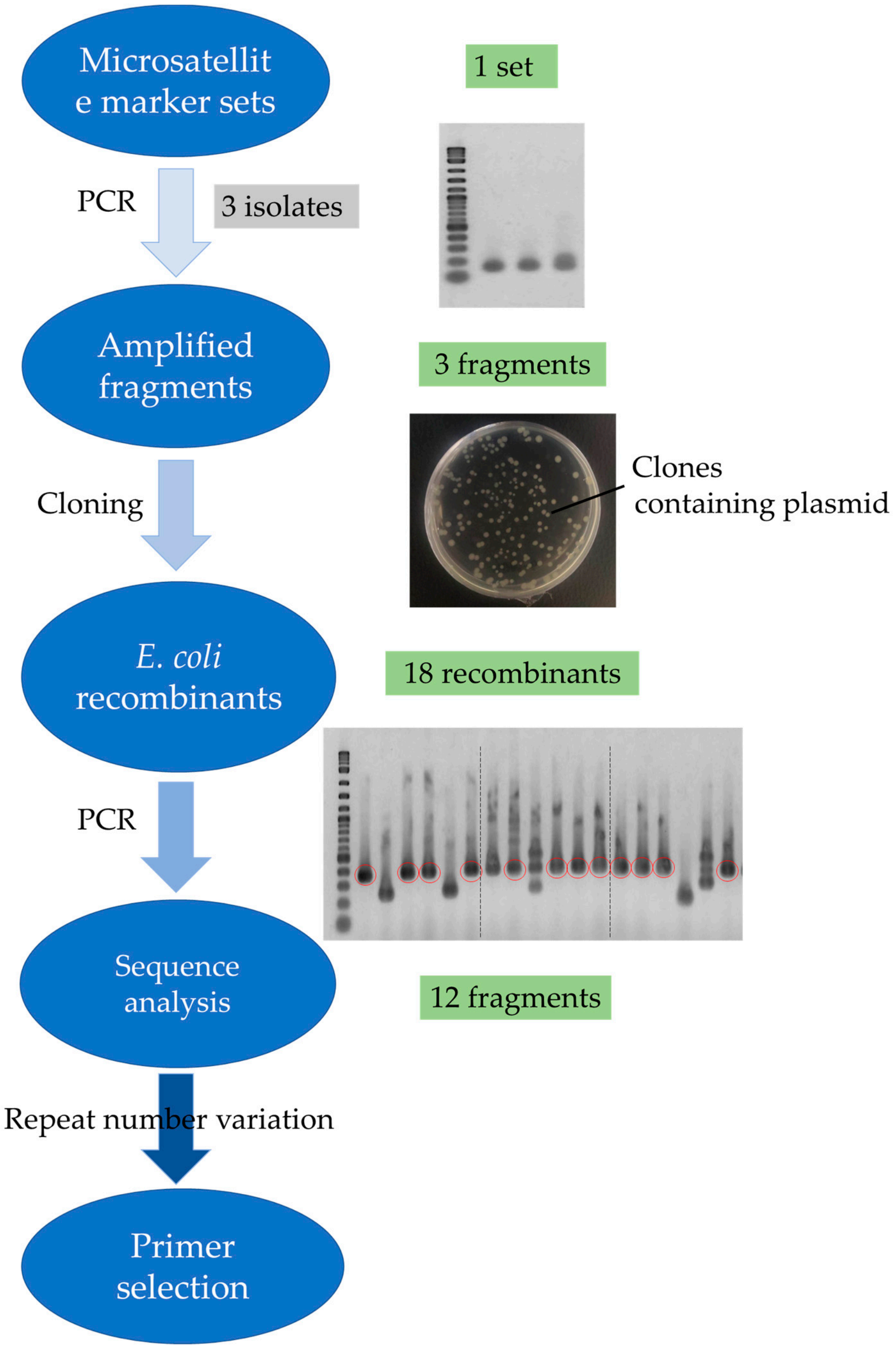

Figure S1. Schematic representation of SSR marker development.

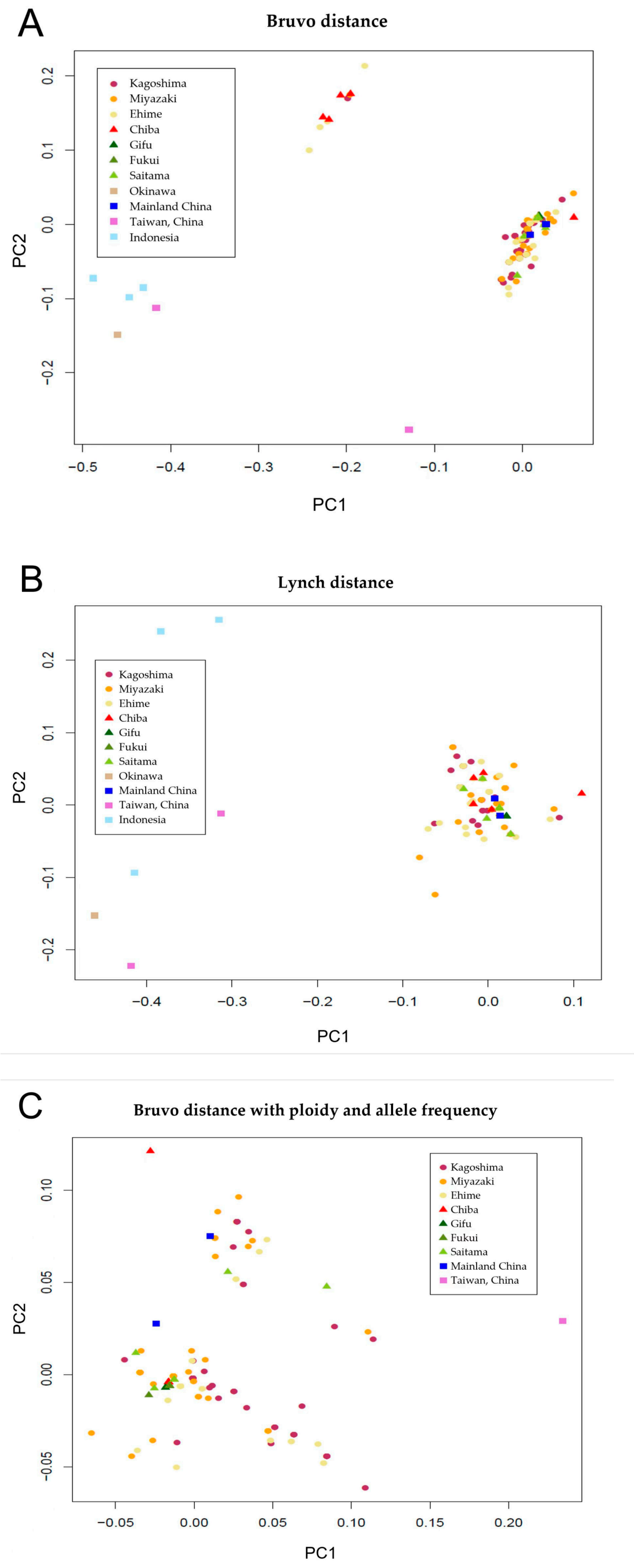

Figure S2. PCA plot generated by the R package "polysat". A: PCA with Bruvo distance for all isolates, B: PCA with Lynch distance for all isolates, C: PCA with Bruvo distance calculated considering ploidy and allele frequency for tetraploid isolates.
